# Supplementary material for: Pentlandite rocks as sustainable and stable efficient electrocatalysts for hydrogen generation
Source: Nat Commun. 2016 Jul 27;7:12269. doi: 10.1038/ncomms12269 (PMC4974457; doi:10.1038/ncomms12269)
Supplement: Supplementary Information — Supplementary Figures 1-26, Supplementary Tables 1-3, Supplementary Notes 1-4 and Supplementary References. [file ncomms12269-s1.pdf]

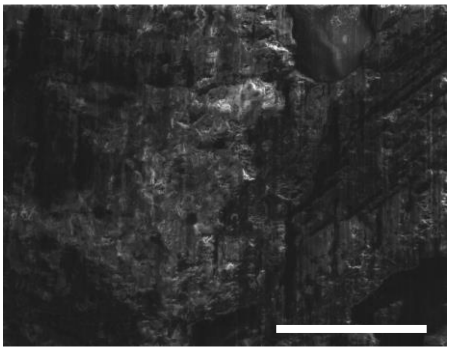

Element	Wt %	At %
O K	41.33	57.18
MgK	11.67	10.63
AlK	2.73	2.24
SiK	26.92	21.22
CaK	11.68	6.45
TiK	0.58	0.27
V K	0.13	0.06
FeK	4.96	1.97
Total	100.00	100.00

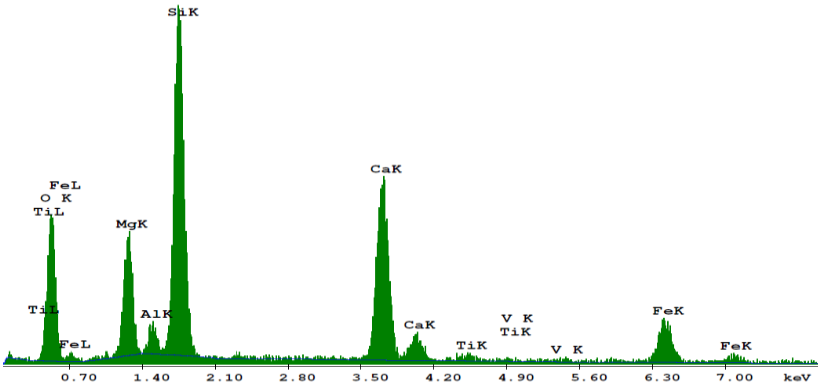

Supplementary Figure 1. SEM images and EDX data of natural pentlandite. SEM image (Scale bar, 200 μm) of natural pentlandite ore along with elemental compositions and corresponding EDX spectra.

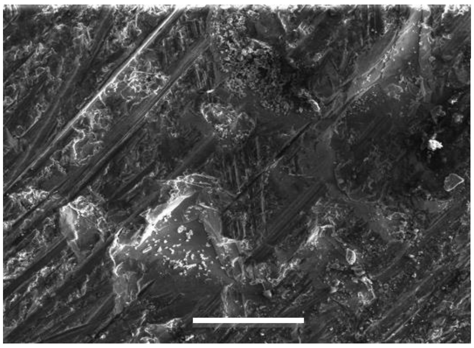

Element	Wt %	At %
C K	25.75	56.68
S K	24.91	20.53
FeK	24.82	11.75
NiK	24.52	11.04
Total	100.00	100.00

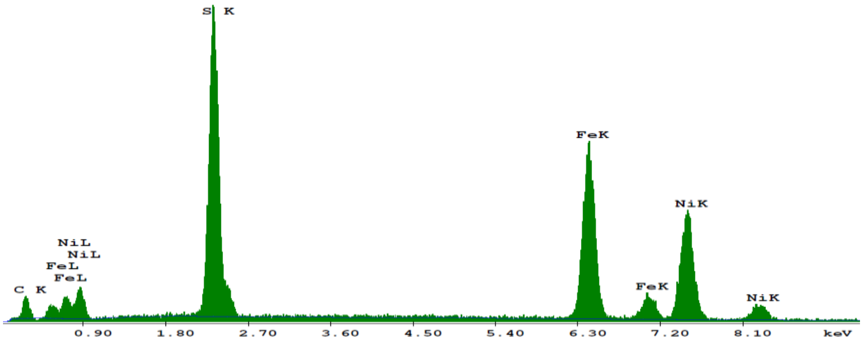

Supplementary Figure 2. SEM images and EDX data of synthetic pentlandite SEM image of as synthesized pentlandite (Scale bar, 100 μm) with the elemental compositions and corresponding EDX spectra.

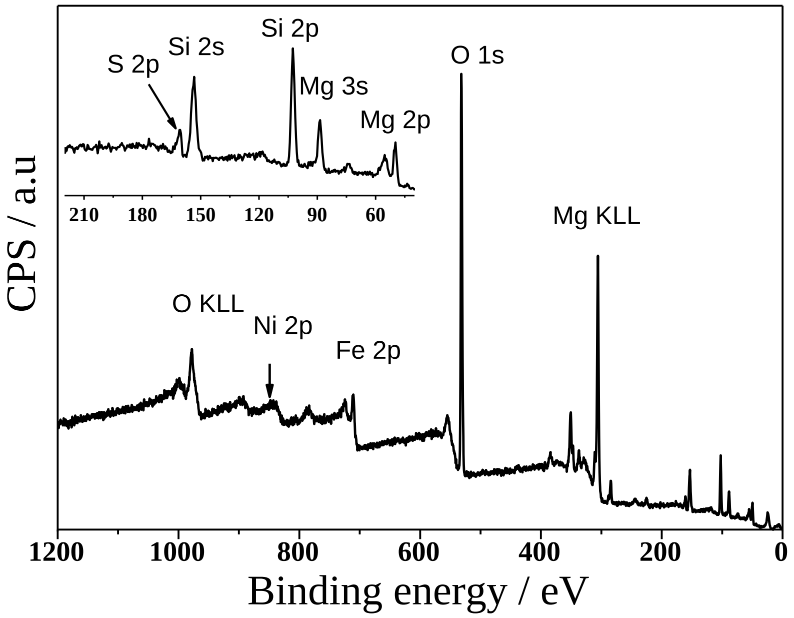

Supplementary Figure 3. X-ray photoemission survey spectra of the natural pentlandite.

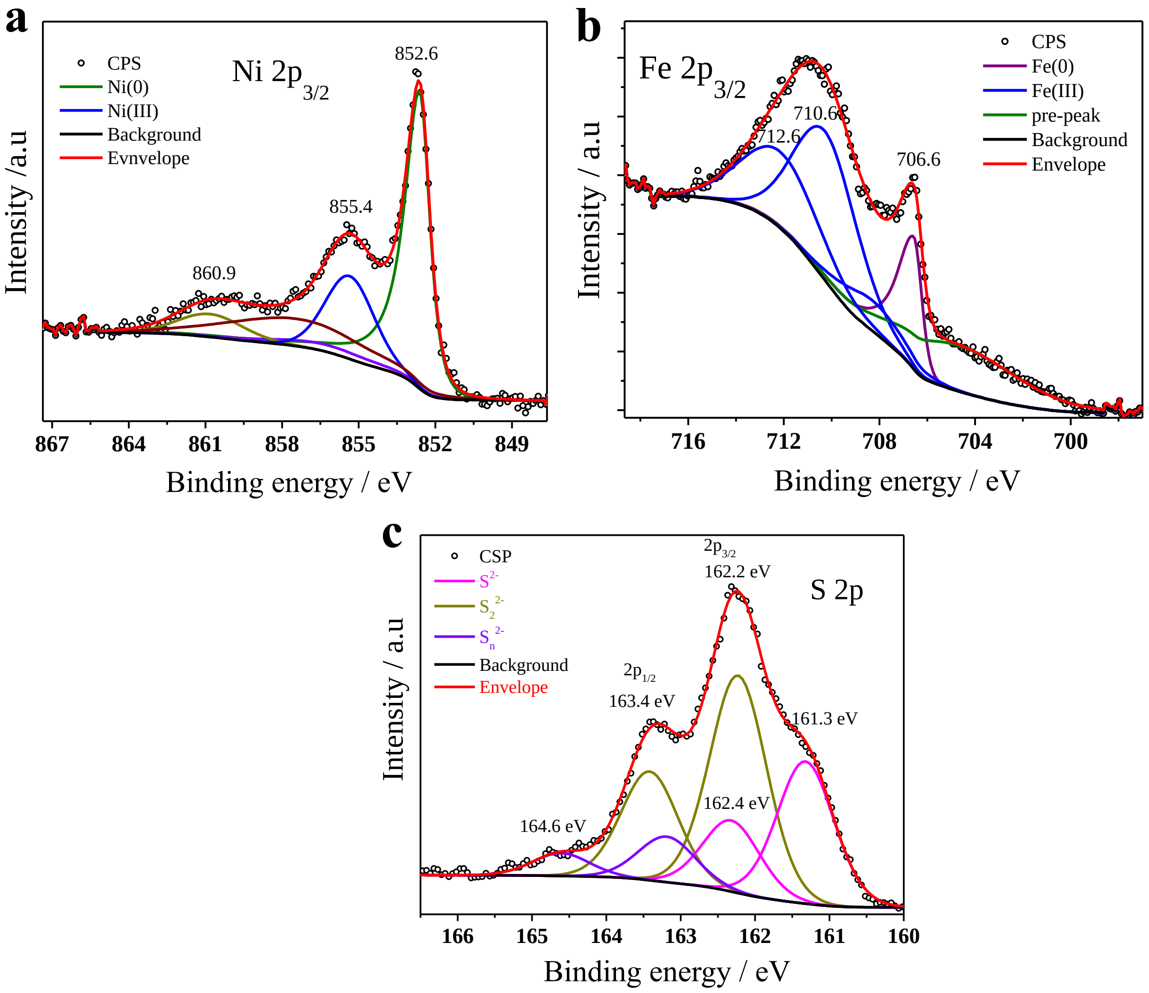

Supplementary Figure 4. XPS data of synthetic pentlandite. Deconvoluted core level XPS spectra of (**a**) Ni *2p*, (**b**) Fe *2p* and (**c**) S *2p* of as synthesized $Ni_{4.5}Fe_{4.5}S_8$ pentlandite. The binding energies of the peak positions are listed in the figures. The C1s peak binding energy (285 eV) was used as reference.

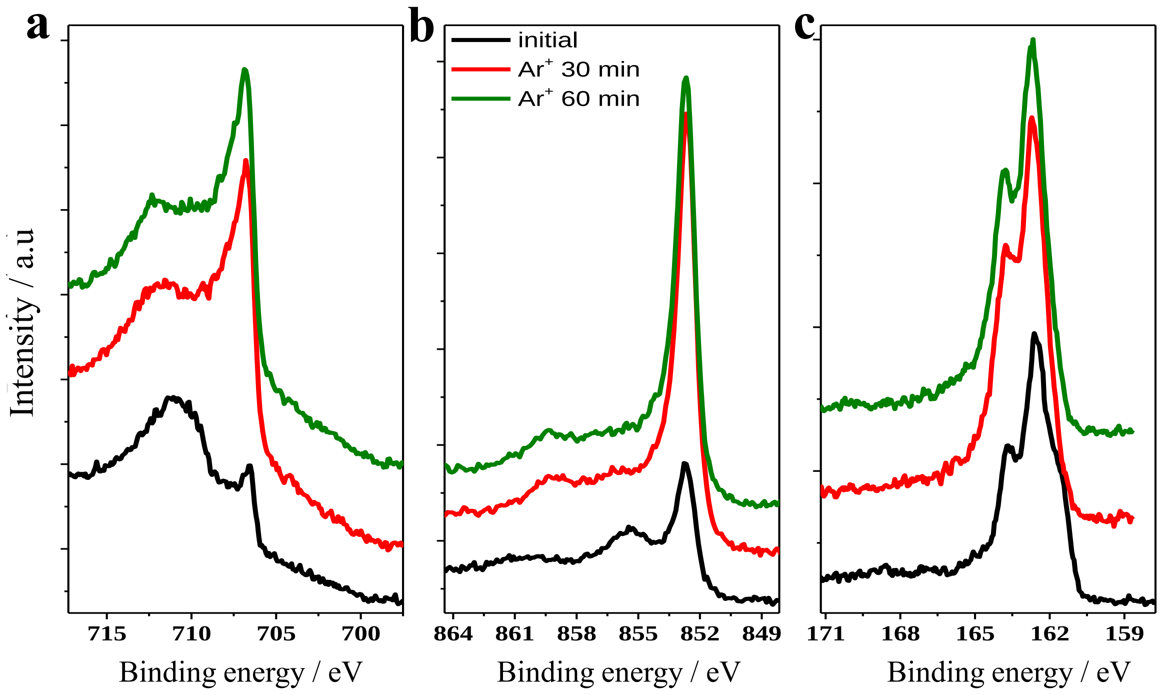

Supplementary Figure 5. Surface cleaning. (**a**) Fe $2p_{3/2}$, (**b**) Ni $2p_{3/2}$ and (**c**) S 2p core level X-ray photoemission spectra of pristine synthetic pentlandite (black) and Ar^+ sputtered synthetic pentlandite after 30 min (red) and 60 min (green) sputtering.

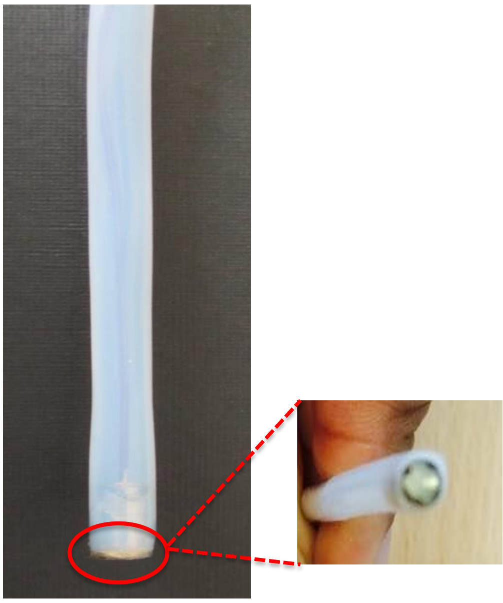

Supplementary Figure 6. Photographic image of the pentlandite rock electrode. The geometric area of the electrode was 0.135 cm^2.

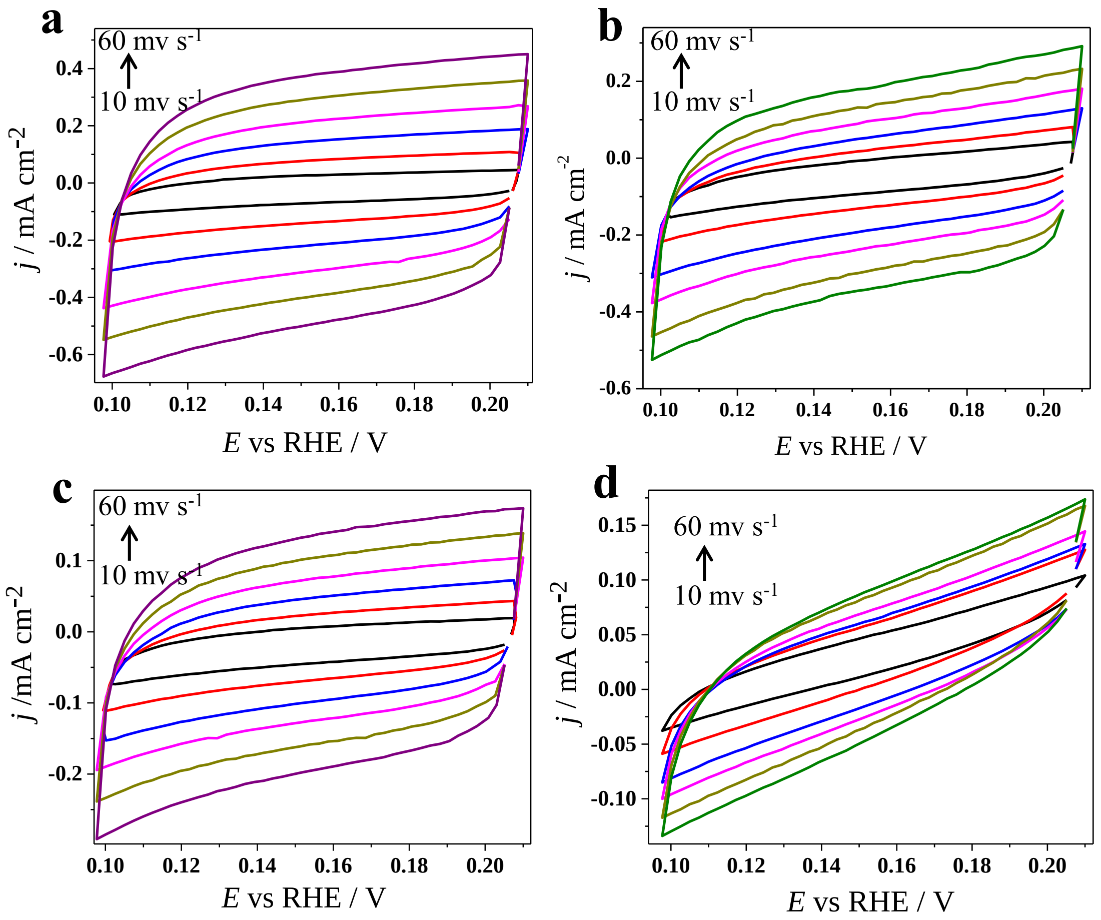

Supplementary Figure 7. Electrochemical surface area. Electrochemical surface area (ECSA) test plots for the studied transition metal sulfides towards the HER in 0.5M H_2SO_4. Cyclic voltammograms of (**a**) NiS_2, (**b**) FeS_2, (**c**) MoS_2 and (**d**) natural rock electrodes at different scan rates varying from 10 mVs^{-1} to 60 mVs^{-1}.

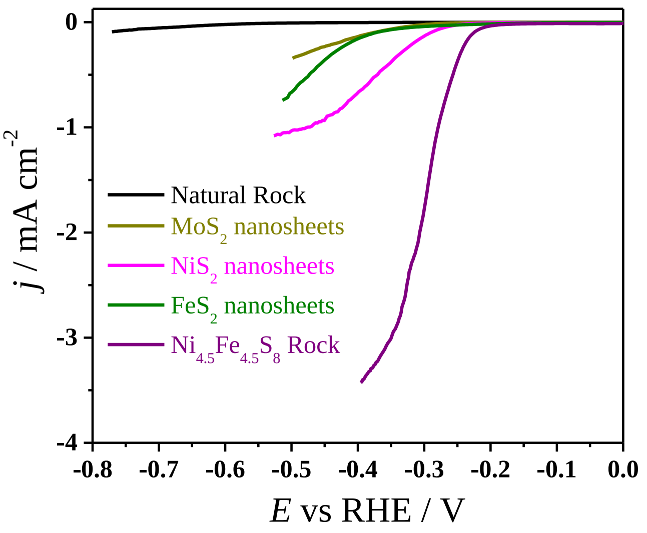

Supplementary Figure 8. Normalized electrochemical surface area voltammograms. Linear sweep voltammograms of the catalysts recorded at a sweep rate of 5 mVs^{-1} in 0.5 M H_2SO_4. The current density is normalized to an electrochemical surface area.

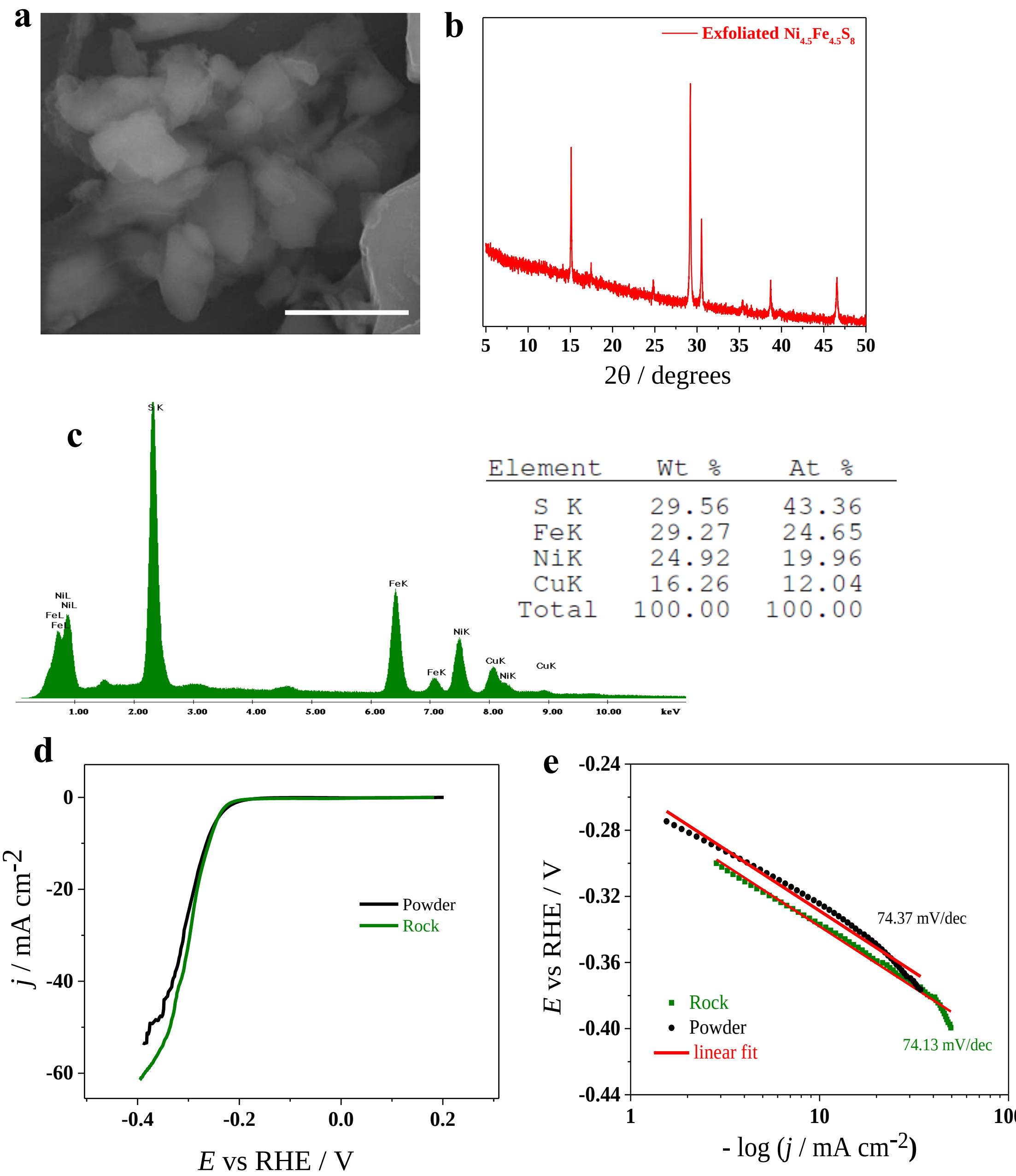

Supplementary Figure 9. Characterization of $Ni_{4.5}Fe_{4.5}S_8$ powder. (**a**) SEM image of exfoliated $Ni_{4.5}Fe_{4.5}S_8$ powder (Scale bar, 1 µm). (**b**) XRD spectra of exfoliated $Ni_{4.5}Fe_{4.5}S_8$. The FWHM of the diffraction peaks is very low; therefore, the size of the exfoliated sheets is expected to be large. (**c**) EDX spectra along with elemental compositions. The Cu peak originated from the sample holder during the SEM/EDX measurements. (**d**) iR corrected RDE linear sweep voltammograms recorded 5 mV s^{-1}. (**e**) Tafel plot. For better comparison, rock and exfoliated $Ni_{4.5}Fe_{4.5}S_8$ were examined under similar conditions.

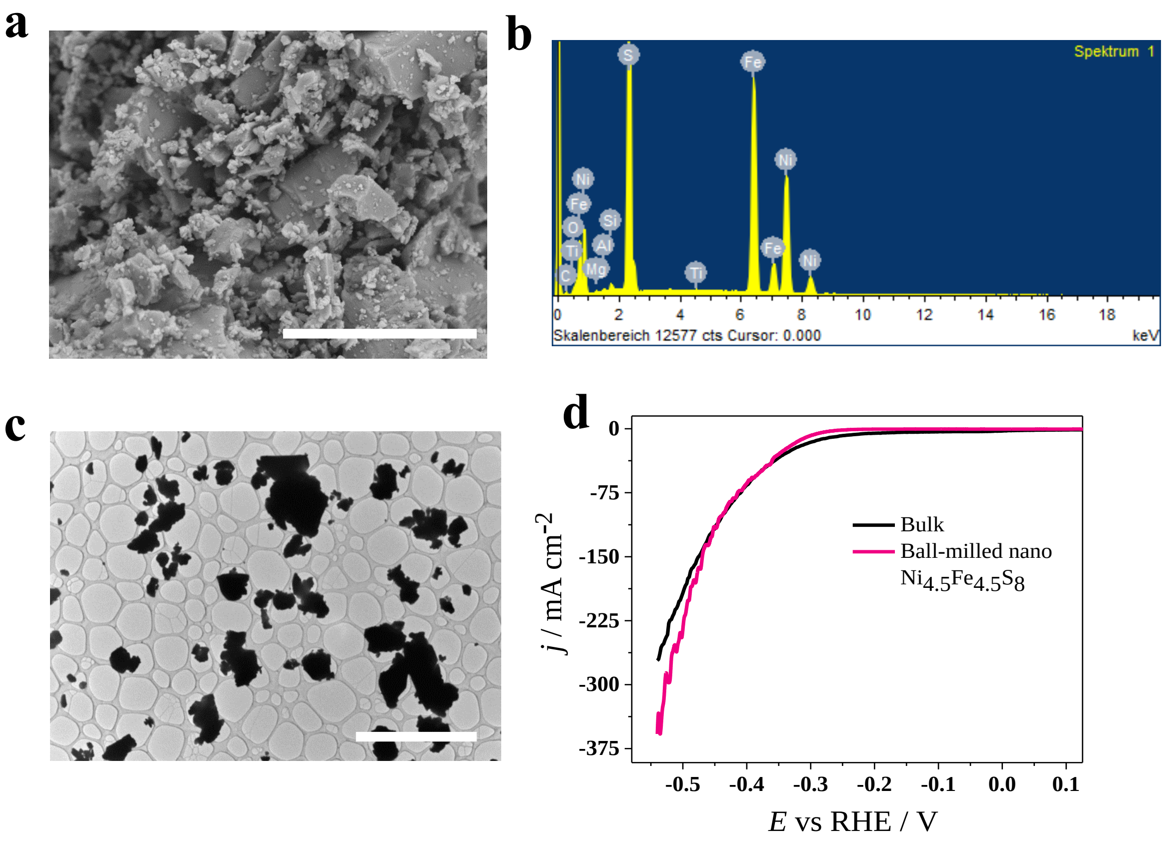

Supplementary Figure 10. Characterization of ball-milled particles. (**a**) SEM image of ball-milled nano-$Ni_{4.5}Fe_{4.5}S_8$ powder (scale bar, 20 μm). (**b**) EDX spectra of ball-milled $Ni_{4.5}Fe_{4.5}S_8$. (**c**) TEM image of ball-milled nano-$Ni_{4.5}Fe_{4.5}S_8$ (scale bar, 5 μm). (**d**) Linear sweep voltammograms recorded 5 mV s^{-1}.

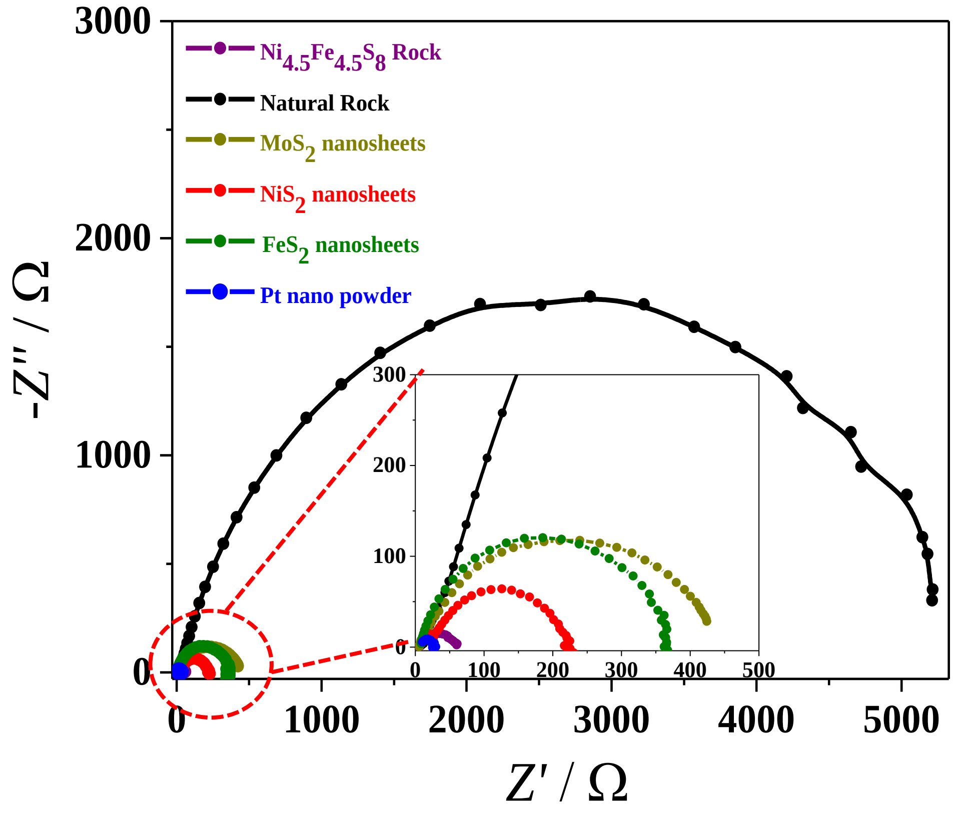

Supplementary Figure 11. Impedance measurements. Nyquist plot of the synthetic $Ni_{4.5}Fe_{4.5}S_8$ rock showing a charge transfer resistance (R_{ct}) of ~57.2 Ω which is smaller than the R_{ct} of NiS_2 (213.2 Ω), FeS_2 (374.6 Ω) nanoparticles as well as MoS_2 (~ 412.4 Ω), and the R_{ct} of natural pentlandite (1.05 kΩ).

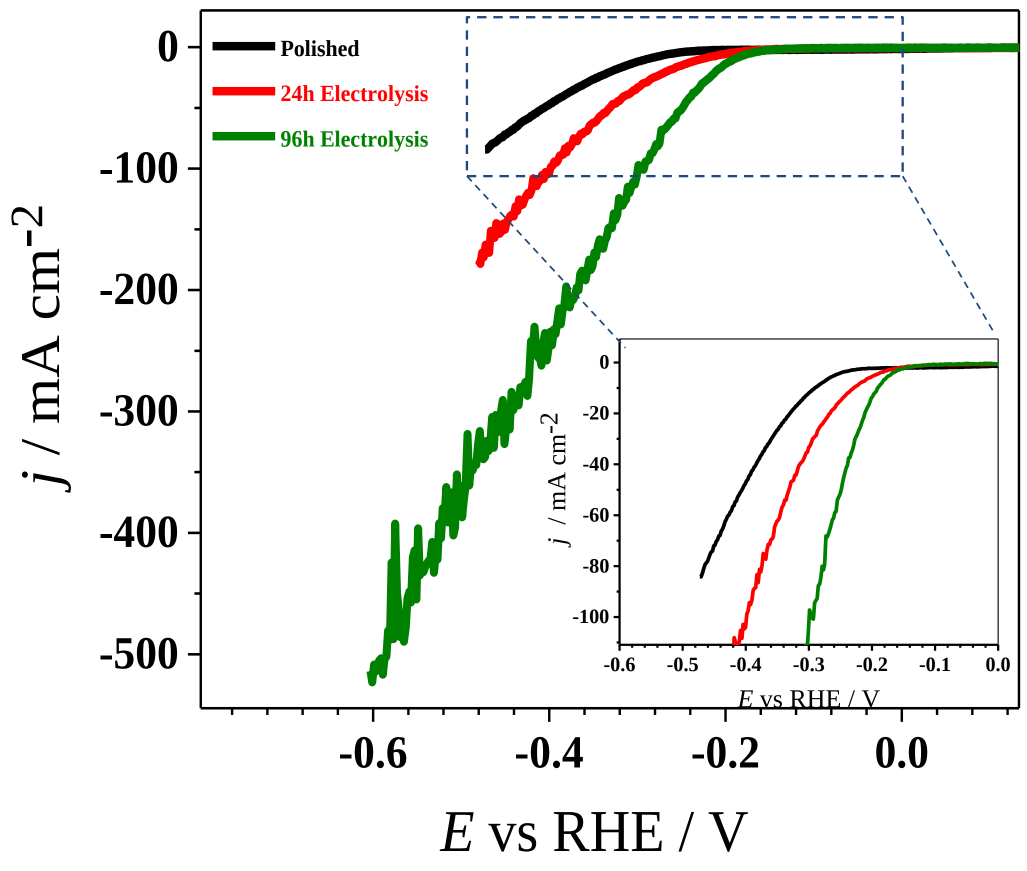

Supplementary Figure 12. Time dependent voltammograms. Linear sweep RDE voltammograms recorded at 5 mV s^{-1} before and after 24 and 96 h electrolysis.

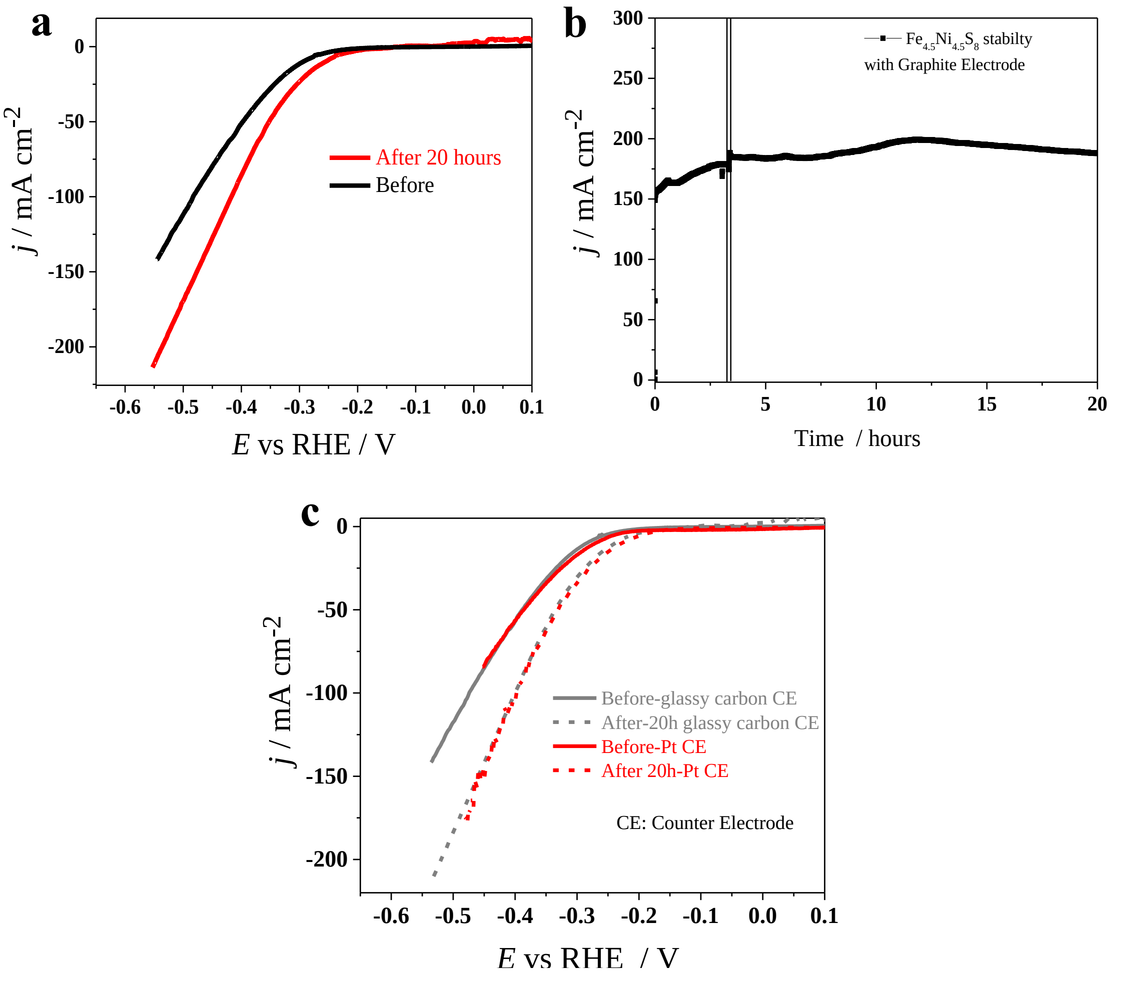

Supplementary Figure 13. Electrochemical durability testing. (**a**) Linear sweep voltammograms recorded at 5 mV s^{-1} before and after 20 h of electrolysis applying a glassy carbon as a counter electrode. (**b**) Long-term current-time plot during 20 h electrolysis versus glassy carbon counter electrode at overpotential of 0.6 V. (**c**) Comparability of HER activity of pentlandite rock using Pt and glassy carbon as the counter electrode.

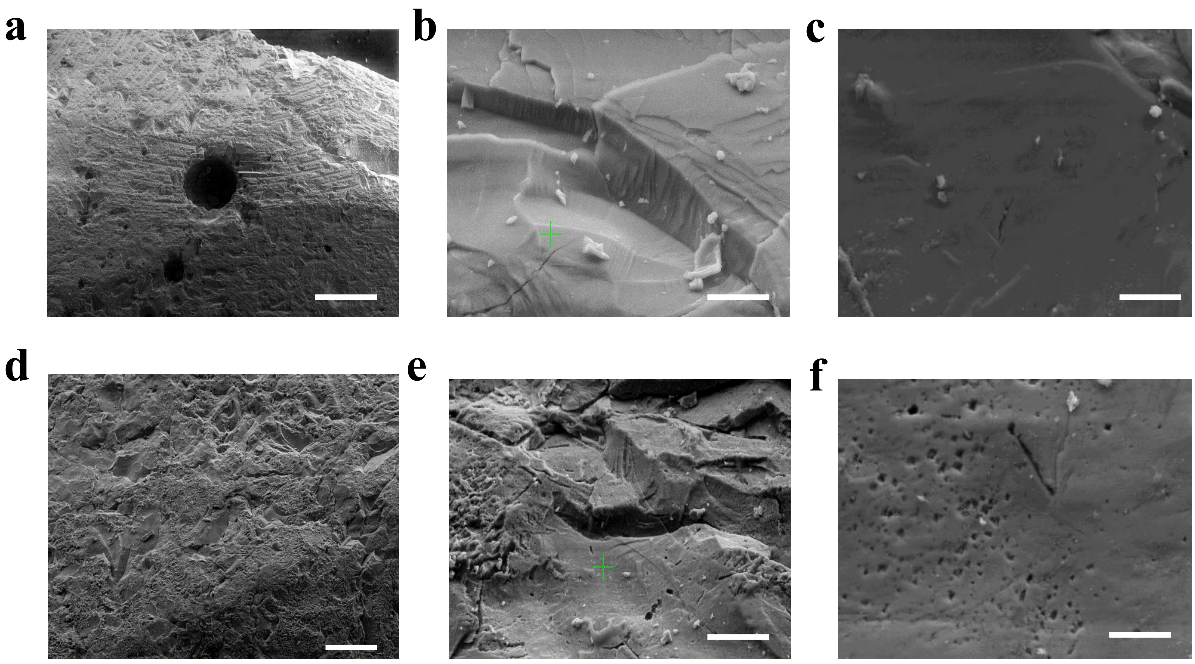

Supplementary Figure 14. Surface alterations during long time measurements. Representative SEM images of the solid pentlandite rock electrode surfaces exposed for zero hour (before) (scale bars, 200 µm (**a**), 10 µm (**b**), 10 µm (**c**)), and after 20 hours of HER (scale bars, 200 µm (**d**), 20 µm (**e**), 10 µm (**f**)).

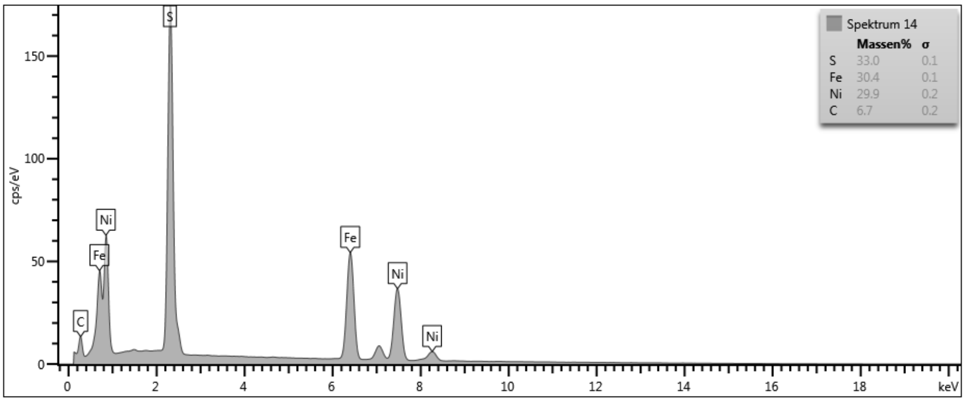

Supplementary Figure 15. EDX of solid pentlandite electrode. EDX spectra of the solid pentlandite rock electrode surface exposed for 20 hours of HER.

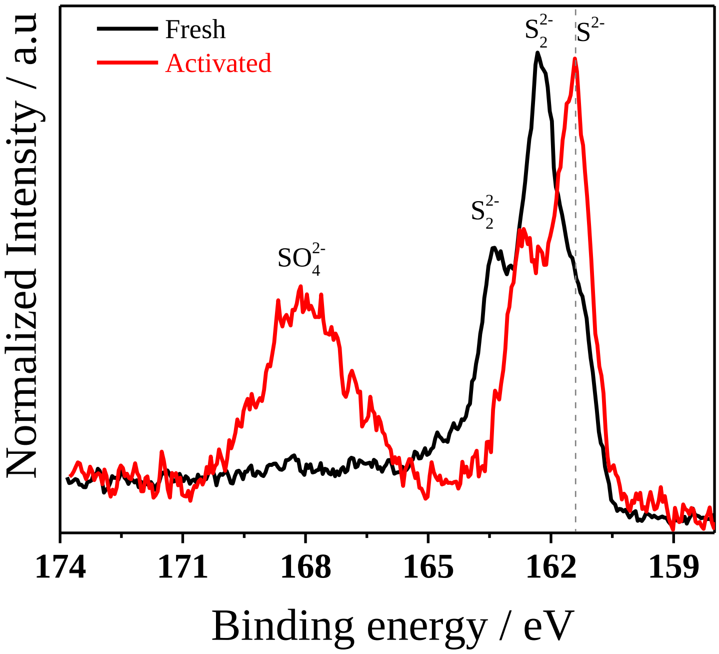

Supplementary Figure 16. XPS data on pentlandite electrodes. Normalized S 2p X-ray photoelectron spectra of solid pentlandite rock before and after 20 h of chronoamperometric electrolysis 0.5 M H_2SO_4. The binding energies are listed in the figure.

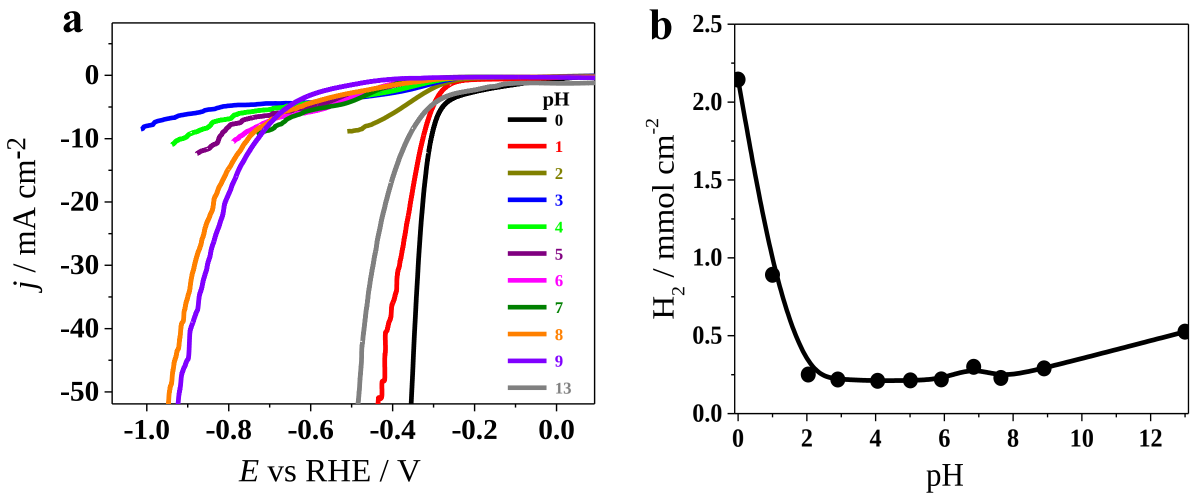

Supplementary Figure 17. pH dependent hydrogen generation. (**a**) HER performance of $Ni_{4.5}Fe_{4.5}S_8$ rock at different pH. Linear sweep voltammograms recorded at a sweep rate of 5 mVs^{-1} in buffer (0.2 M $B(OH)_3$, 0.05 M citric acid and 0.1 M Na_3PO_4) at different pH values. (**b**) Turnover frequency (TOF) of the pentlandite-based HER catalyst at different pH values for 1 hour of electrolysis. Notably, the TOF is highest at low pH values and drops to ~1/5 of its original value between pH 2 and 9. At higher pH values the TOF increases again. Evidently, the higher the ionic strength the higher is the TOF of the HER. Similar results were observed when e.g. NaCl, Na_2CO_3 or Na_3PO_4 were added to the electrolyte solution. The TOF was calculated according to TOF = $n(H_2)/\{A(cat) \times t\}$ with n is the amount of substance, A is the catalyst area and t is time

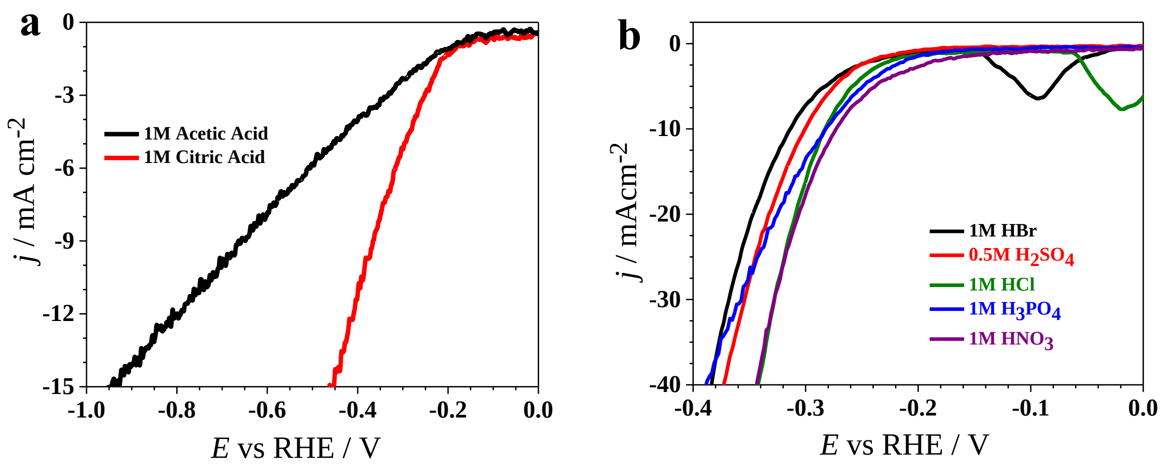

Supplementary Figure 18. **Acid dependent hydrogen evolution.** HER performance on $Ni_{4.5}Fe_{4.5}S_8$ rock in different acids. (**a**) and (**b**) Linear sweep voltammograms recorded at 5 mV s^{-1} in different acids.

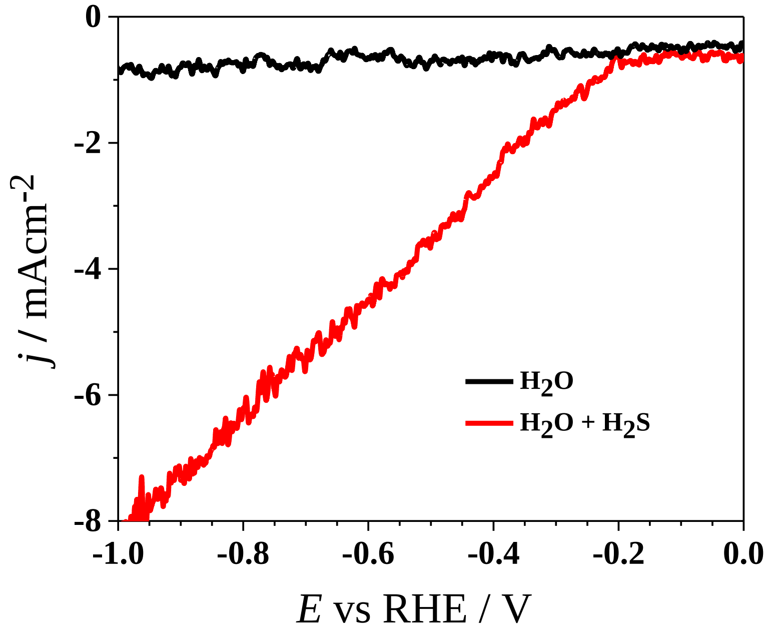

Supplementary Figure 19. The application of H_2S as proton source for HER.

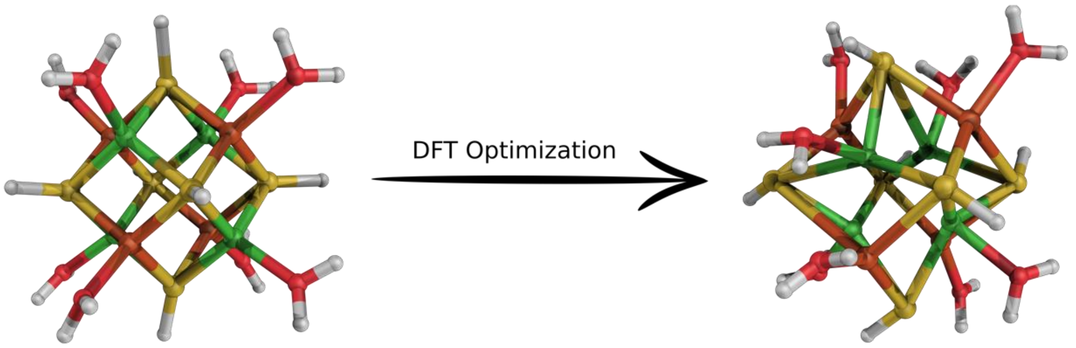

Supplementary Figure 20. Cluster deformation. Deformation of a $H_6Ni_4Fe_4S_6(H_2O)_8^{2+}$ cluster during the transition from embedded to exposed conditions (color scheme: green: nickel; white: hydrogen; red: oxygen; brown: iron; yellow: sulfur).

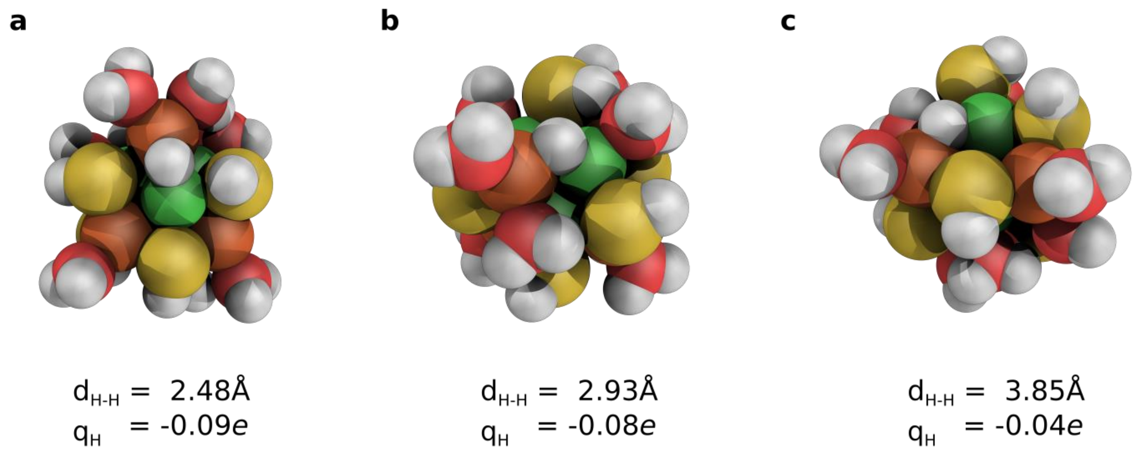

Supplementary Figure 21. Spontaneous protonation. Clusters with multiplicity 17 protonated at different sites and corresponding charges of the hydride species that bridging between two metal atoms (**a**, **b**, **c**). Note that structure (**a**) corresponds to structure (**a**) in Figure 5 (color scheme: green: nickel; white: hydrogen; red: oxygen; brown: iron; yellow: sulfur).

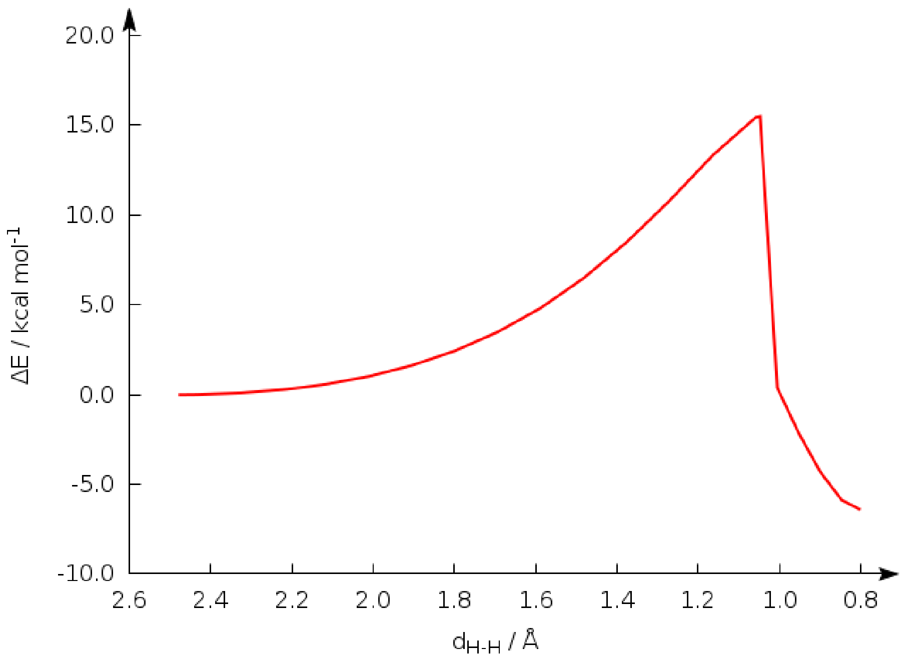

Supplementary Figure 22. Hydrogen formation. Potential energy scan along the shortest H-H distance, used to force the formation of H_2 on the cluster.

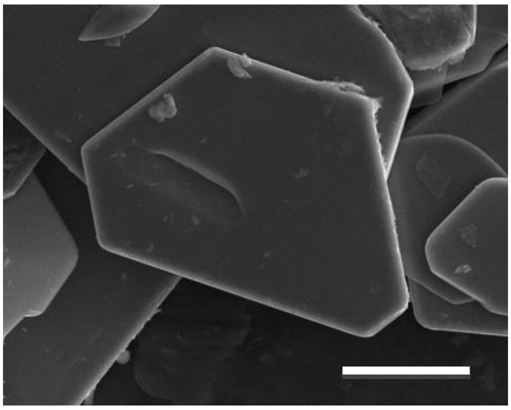

Element	Wt %	At %
MoL	62.71	37.11
S K	37.29	62.89
Total	100.00	100.00

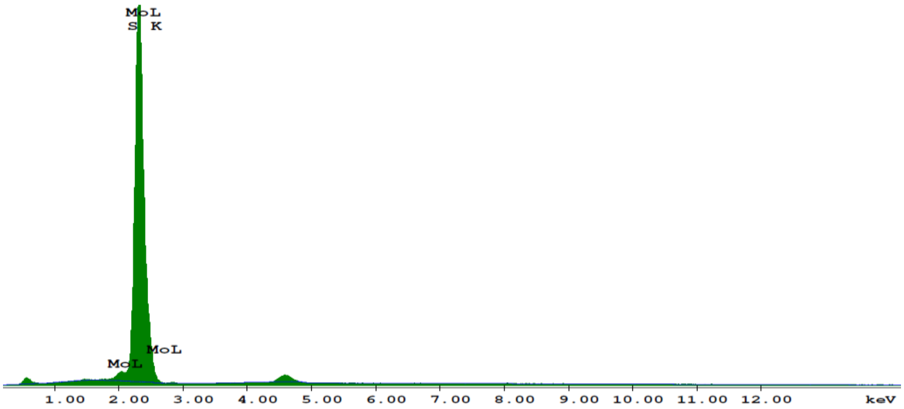

Supplementary Figure 23. SEM, EDX data of MoS_2. SEM image and EDX spectra of the as synthesized MoS_2 bulk crystals along with elemental compositions (scale bar, 20 µm).

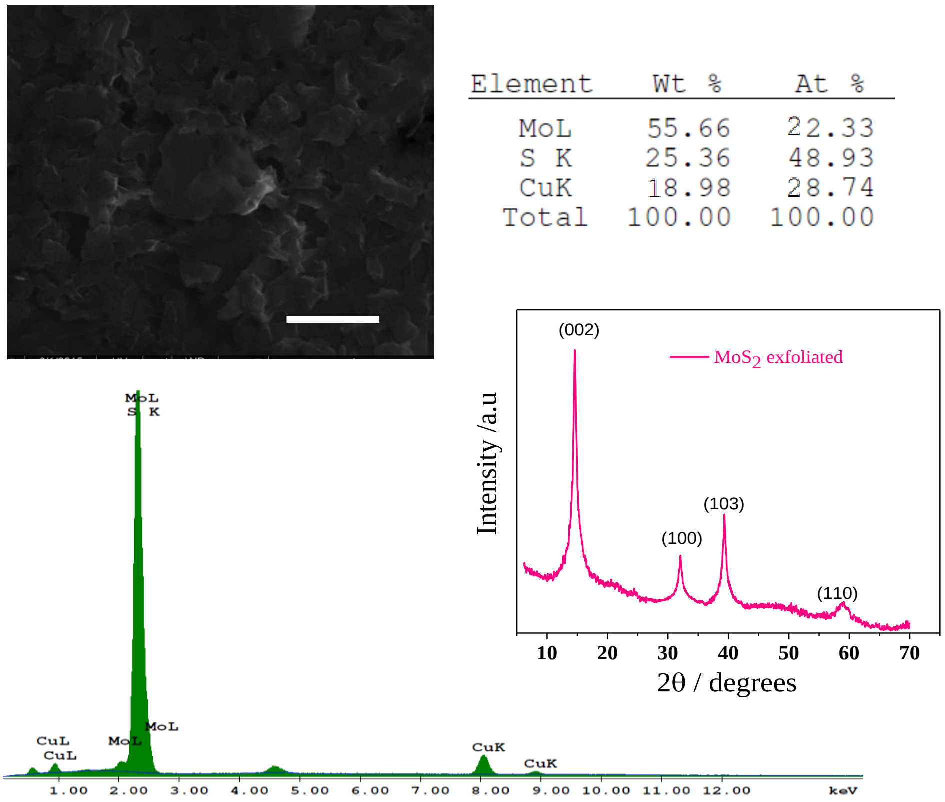

Element	Wt %	At %
MoL	55.66	22.33
S K	25.36	48.93
CuK	18.98	28.74
Total	100.00	100.00

Supplementary Figure 24. SEM, EDX and XRD data of exfoliated MoS_2. SEM image and EDX spectra of the as exfoliated 2H-MoS_2 nanosheets along with elemental compositions and XRD spectra (JCPDF # 37-1492) (scale bar, 1 μm). The intense Cu peak originated from the substrate.

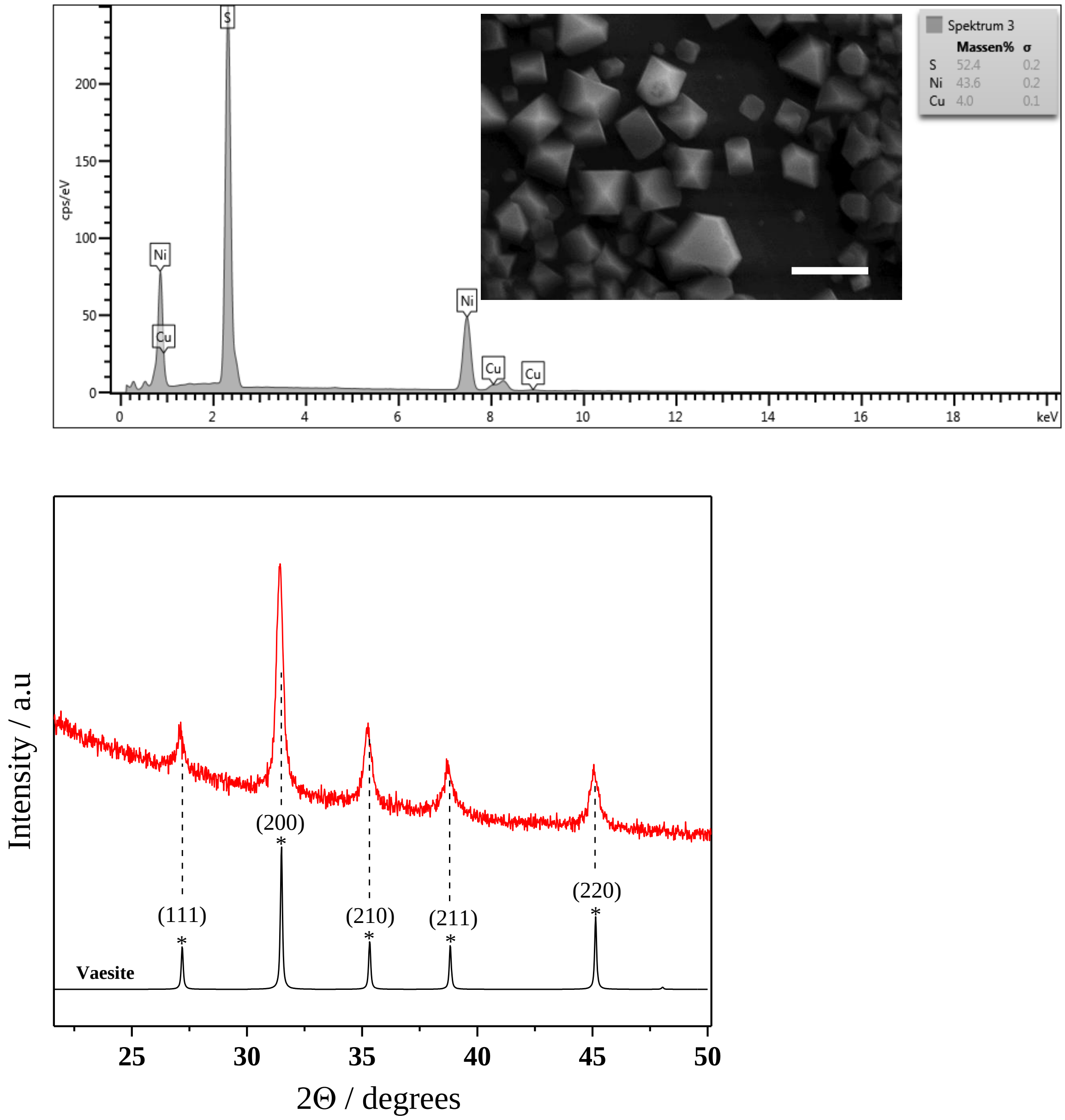

Supplementary Figure 25. SEM, EDX data of NiS_2. SEM image and EDX spectra of the as NiS_2 nanocrystals along with elemental compositions and XRD spectra. (scale bar, 1 μm) The Cu peak in EDX spectra originated from the substrate.

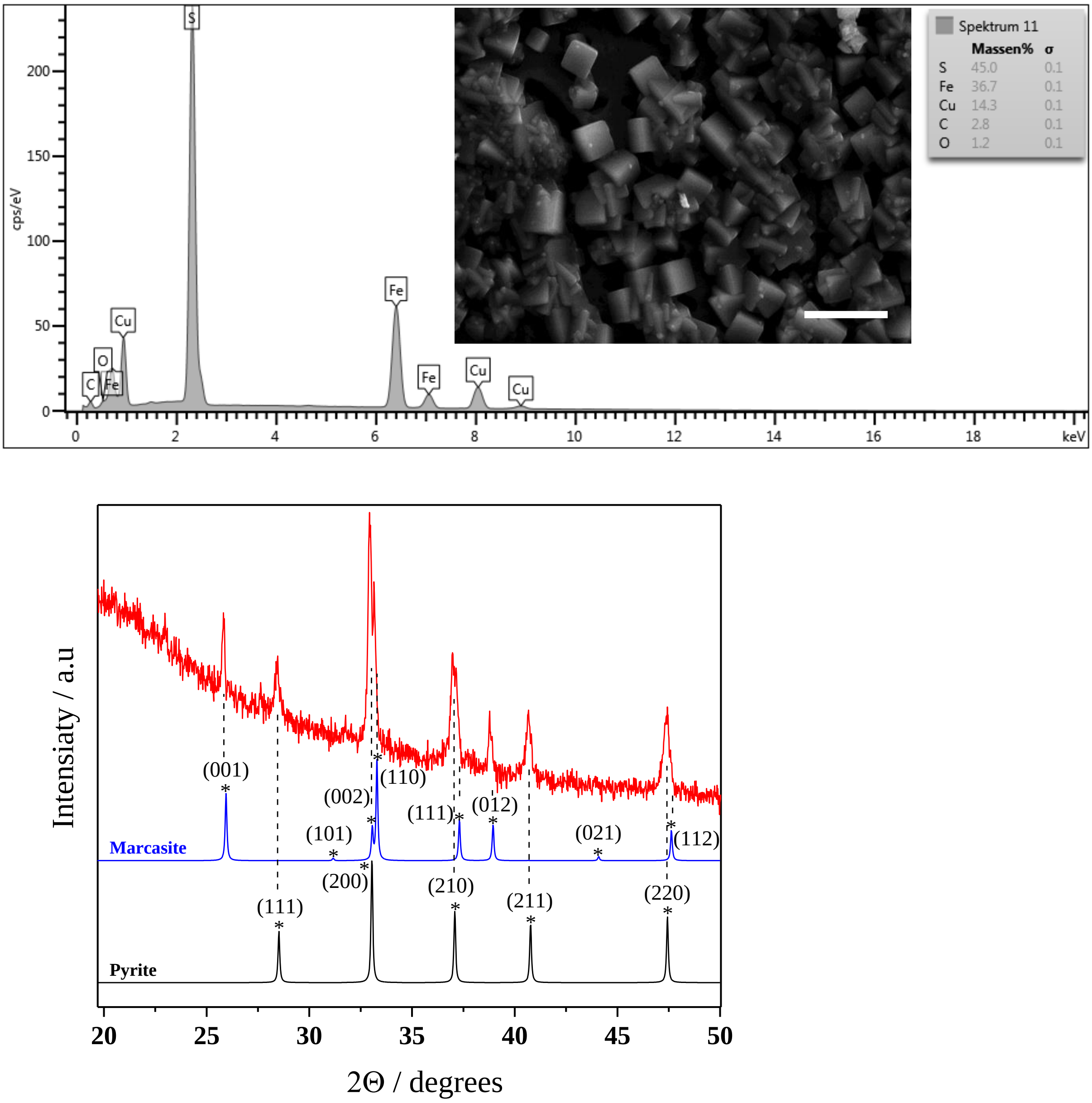

Supplementary Figure 26. SEM, EDX data of FeS_2. SEM image and EDX spectra of the as FeS_2 nanocrystals along with elemental compositions and XRD spectra (scale bar, 2 μm). The intense Cu peak originated from the substrate.

Supplementary Table 1. XPS-derived composition of synthetic pentlandite before and after sputtering with Ar^+ for 30 and 60 min.

Sample	Fe(0):Fe(III)	Ni(0):Ni(III)	Fe:S	Ni:S
Pristine	0.24	1.51	0.23	0.19
Ar^+ 30 min	0.71	8.83	0.43	0.26
Ar^+ 60 min	0.74	8.76	0.42	0.28

Supplementary Table 2. Comparison of literature known and herein reported HER catalysts.

Material	Morphology	E_{RHE} [mV]	j_{Cat} [mA cm^{-2}]	Resistance Ω	Faradaic efficiency [%]	H_2 produced [mmol h^{-1} cm^{-2}]
Natural pentlandite[a]	Rock	~500	10	1050	---	---
Synthetic pentlandite[a]	Rock	280-190<br>~ 800	10<br>≥ 650	57.2	94	2.14
FeS_2[a]	Nano-particles	400	10	374.6	---	
NiS_2[a]	Nano-particles	315	10	213.2	---	---
MoS_2[a]	Nanosheets	374	10	412.4	---	---
Pt[a]	Nanopowder	38	10	17.5	---	---
MoC_x[1]	Nano-octahedrons	~142	10	---	---	---
MoC_x[1]	Nano-particles	230	1	---	---	---
$NiMoN_x/C$[2]	Nanosheets	200	3	---	---	---
MoN/C[2]	Nanosheets	350	3	---	---	---
MoS_2/Au[3]	Nano-particles	150	0.92	300-400	---	---
MoS_2[4]	Nano-particles	190	2	---	---	---
WS_2[5]	Nanoflakes	180	10	---	---	---
WS_2/graphene[6]	Nanosheets	280	10	---	---	---
FeP[7]	Nanosheets	200	10	---		---
α-INS[8]	Nanosheets	105	10	63.2	---	---
$CoSe_2$[9]	Nano-particles	200	10	---	---	---
CoSe[10]	Nanobelts	125	10	---	---	---
Ni_2P[11]	Nano-particles	180	20	---	~100	7.4
Pt[12,12]	Nano-particles	1200	600	---	96	11
		90	10	---	---	---
NiS_2[13]	Nanofilms	230	1	---	---	---
FeS_2[13]	Nanofilms	217	1	---	---	---

[a] Material investigated within this study.

Supplementary Table 3. **DFT-calculated energies of optimized $H_6M_8S_6(H_2O)_8^{2+}$ cluster with different Fe/Ni ratio and spin state**

Cluster	Multiplicity	E(rel), kcal mol^{-1}
$H_6Fe_4Ni_4S_6(H_2O)_8^{2+}$	1	41.3
	3	33.2
	9	16.7
	11	12.0
	13	3.0
	15	1.0
	17	0.0
	19	5.3
	21	19.1
$H_6Fe_8S_6(H_2O)_8^{2+}$	1	57.3
	3	51.2
	23	30.1
	25	18.5
	27	0.0
	29	13.1
	31	26.5
$H_6Ni_8S_6(H_2O)_8^{2+}$	1	14.0
	3	8.6
	7	7.6
	9	0.0
	11	8.1
	13	30.6

Supplementary Note 1

Supplementary Fig. 5 shows X-ray photoemission (XP) spectra of pristine synthetic pentlandite as well as after 30 and 60 minutes of Ar^+ sputtering. Both Fe and Ni appear severely oxidized in pristine samples, which is evident from peaks at 711.6 and 855.4 eV (black curves on Supplementary Fig. 5a and b, correspondingly) reflecting the presence of oxide metal species. The peaks at 706.5 and 852.7 eV can be attributed to metallic states which are in good agreement with values reported for NiS[14] and FeS_2[15] compounds. Detailed deconvolution of XP spectra of first row transition metals is particularly challenging due to strong multiplet separation[16]. In particular, Ni 2p XP lines from oxide- and hydroxide species are described by envelopes of 5 and 7 peaks correspondingly[17]. Not aiming to assess detailed information on higher oxidation states of both Fe and Ni, we have used three pseudo-Voigt profiles for iron oxides and two for oxidized nickel. The latter is most likely represented by Ni(III) (e.g. NiOOH) species since no intense shake-up structure above 860 eV characteristic for Ni(II) is observed. Relatively small width of peaks attributed to oxidized iron and absence of intense shake-up satellites suggests presence of Fe(III) species[18]. For metallic states asymmetric peak shapes were used following the procedure suggested by Biesinger and co-workers[16]. Fe(0):Fe(III) and Ni(0):Ni(III) ratios obtained from deconvolution XP spectra are correspondingly 0.23:1 and 1.51:1 for pristine synthetic pentlandite (see Supplementary Table 1). After 30 min of Ar^+ sputtering the amount of reduced iron increased reaching 73.4 at.%, while only a small amount of oxidized Ni is observed. The latter is hard to estimate though and the value strongly depends on the way the spectral background is constructed. The highest was as low as 10 at.%. S 2p core level spectrum of pristine pentlandite (Supplementary Fig. 5c, black curve) which can be deconvoluted into three doublets (see Supplementary Fig. 4 for detailed plot). The S 2p spectrum of a disulphide (S_2^{2-}) ion exhibited spin orbit doublet at

162.2 eV and 163.4 eV and sulphide ion (S_2^{2-}) the lower binding energy of 161.3 eV and 162.4 eV, results agrees with the literature values for FeS_2 and NiS, respectively[1,2]. Polysulphide (S_n^{2-}) ion showed doublet at 164.6 eV and 163.1 eV. It is also worth noting, that no signals related to oxidized sulfur were observed around 169 eV. The following Ar^+ sputtering for 30 min did not change neither the chemical state nor the near surface composition of the sample.

Supplementary Note 2

We investigated an approximately 4 x 1 x 0.5 cm solid pentlandite rock for chronoamperometric electrolysis for 20 h applying a glassy carbon plate as counter electrode in 0.5 M H_2SO_4. The analyzed area is the electrode part used for the HER that fully dipped into H_2SO_4. We explored the morphology as well as chemical composition of the rock by SEM/EDX and XPS. Notably, although the morphology is slightly rougher, the overall composition of the material remains unaltered (Supplementary Figure 14 and 15). Since the EDX is not a surface sensitive method and allows penetration of up to 3 µm, we performed additional XPS measurements. For fresh sample, S 2p spectrum of a disulphide (S_2^{2-}) ion exhibited spin orbit doublet at 162.2 eV and 163.4 eV and sulphide ion (S_2^{2-}) the lower binding energy of 161.3 eV and 162.4 eV (Supplementary Figure 16). It is believed, however, that after electrolysis of 20 h the S 2p spectrum of a sulphide ion (S^{2-}) intensity is raised up suggesting the rock-material loss surficial disulfide-type sulfides from the catalyst surface resulting in sulfur vacancies which in turn improved the reactivity of the exposed metal active sites and the HER performance[19]. The peak at higher BE of 168.2 eV is attributed to SO_4^{2-} species and obviously originates from deposition of SO_4^{2-} from the electrolyte (Supplementary Figure 16).

Supplementary Note 3

The crystal structure of pentlandites can be best described as a M_8S_6 cuboctahedra (MSCOs) interconnected by tetrahedrally bound sulfur and additional in-plane metal atoms. Hence, an intersecting plane, forming a surface of a crystal will dissect these MSCO units, making them a reasonable model for a surface exposed fragment. We assumed the $H_6M_8S_6(H_2O)_8^{2+}$ polysulfidometallate cation to be an appropriate model for the smallest reactive unit in acidic media. This stems from the close resemblance of the structural motif and the inherent short M-M contacts (2.507 Å) that are of particular interest as potential reaction centers (Supplementary Figure 20). In addition, the assumed sulfide/oxide bleaching under reductive conditions affords surface accessible metal atoms that are most likely interacting with solvent molecules. Independent of the initial Fe/Ni distribution at the different metal positions within the cuboctahedral cluster, the ratio of Fe/Ni as well as the spin state, the $H_6M_8S_6(H_2O)_8^{2+}$ cluster loses its ordered shape (Supplementary Figure 20 and Supplementary Figure 21). One can expect that similar to the deformation of the $H_6M_8S_6(H_2O)_8^{2+}$ cluster, the surface exposure in the bulk material leads to comparable structural changes. Protons bound to sulfur atoms subsequently are declined from tetrahedral vertices indicating a strongly unsaturated character of sulfur-containing groups on the surface. Most probably these sulfur atoms tend to form strong H-bonds with water molecules. In agreement to the Mössbauer data of $Ni_{4.5}Fe_{4.5}S_8$ (Figure 2b), and as expected for a conducting material, calculations show that the high-spin states are much more stable than the low-spin ones (Supplementary Table 3), indicating multiple degenerate levels at the Fermi level.

In order to locate a resting state for the potential hydrogen formation, optimizations of systems protonated at different water molecules (essentially replacing it by a hydronium ion) were performed. In all cases, the hydrogen atom was transferred from the hydronium ion to a metal atom leading to a hydride between a nickel and iron atoms. For the case with the largest

negative charge on the hydrogen, a reaction path for the hydrogen formation was determined by a series of constrained geometry optimizations, using the shortest H-H distance as a reaction coordinate. During this process another proton that was originally bonded to a sulfur atom is pulled towards the hydride and leads to the formation of H_2, which is bonded non-classical to the nickel atom with a d_{H-H} distance of 0.801 Å only slightly above the gas phase value at the same level of theory (d_{H-H} = 0.761 Å). The first order transition-state, located by Eigenvector following, reveals an activation barrier of 15.5 kcal mol^{-1}. As expected, the formation of H_2 is an exothermic process (ΔE = -6.4 kcal mol^{-1}). The relevant structures during the H_2 formation are shown in Figure 5, the potential energy curve along the H-H coordinate is shown in Supplementary Figure 22. The results for the model system calculations corroborate the potential of pentlandite to electrocatalyse the HER with a mechanism, strikingly similar to the biological hydrogenase.

Supplementary Note 4

All quantum chemical calculations have been performed on the hybrid density functional theory level using the PBE0 [20–22] functional. All electron basis sets of double-zeta split-valence quality [23] have been used (def2-SVP). The screening of spin states, summarized in Supplementary Table 3, has been performed with the ORCA program package[24]. Here, auxiliary basis sets for the Coulomb-fitting were used. For each multiplicity the cluster was re-optimized, leading to slightly different structures with a less closed structure, as indicated in Supplementary Figure 20. Clearly, high spin–states with multiple unpaired electrons are energetically preferred.

For the investigation of the hydrogen formation reaction coordinate we chose the lowest energy S=17 state and kept it constant during the reaction. These calculations were performed on the same level of theory using the TURBOMOLE V6.6 program package[25,26]. The dispersion correction D3 according to Grimme was added[27]. The Resolution of Identity (RI) approximation[28–30] and the MARIJ method[31] was used. As explained in the main paper, the optimized cluster was protonated at three different water sites and relaxed. The resulting structures are shown in Supplementary Figure 21. In all cases the hydrogen is transferred to a metal atom and becomes negatively charged (hydridic), as shown by the Mulliken charges for these species given also in Supplementary Figure 21. The stationary point with the highest negative charge and the shortest distance to a H(-S) was chosen as the starting point for the scan along the reaction coordinate (see Supplementary Figure 22). By sequential optimizations with the H-H distance fixed to values, reduced by 0.106 Å in each step, the energy was scanned along this coordinate (Supplementary Figure 22). After the maximum slightly above 1.0 Å the system reorganizes and forms a non-classical hydrogen complex with a hydrogen molecule bound to the nickel atom.

At the point highest in energy of the scan, the second derivative of the energy with respect to the atomic coordinates (Hessian matrix) was computed using the *aoforce* program of TURBOMOLE. The first order transition state of the hydrogen formation was in turn located by Eigenvector following. The nature of the transition state was analyzed by a further computation of the Hessian matrix at this stationary point. The normal mode analysis gave a single mode with negative curvature and an imaginary frequency of i975 cm^{-1}.

References

1. Wu, H. B., Xia, B. Y., Yu, L., Yu, X.-Y. & Lou, X. W. (David). Porous molybdenum carbide nano-octahedrons synthesized via confined carburization in metal-organic frameworks for efficient hydrogen production. *Nature Commun.* 6:6512 doi: 10.1038/ncomms7512 (2015)..

2. Chen, W.-F. *et al.* Hydrogen-Evolution Catalysts Based on Non-Noble Metal Nickel–Molybdenum Nitride Nanosheets. *Angewandte Chemie International Edition* **51,** 6131–6135 (2012).

3. Troppmann, S. *et al.* Enhanced Photocatalytic Hydrogen Production by Adsorption of an [FeFe]-Hydrogenase Subunit Mimic on Self-Assembled Membranes. *European Journal of Inorganic Chemistry* **2016,** 554–560 (2016).

4. Kibsgaard, J., Chen, Z., Reinecke, B. N. & Jaramillo, T. F. Engineering the surface structure of MoS2 to preferentially expose active edge sites for electrocatalysis. *Nat Mater* **11,** 963–969 (2012).

5. Cheng, L. *et al.* Ultrathin WS2 Nanoflakes as a High-Performance Electrocatalyst for the Hydrogen Evolution Reaction. *Angewandte Chemie International Edition* **53,** 7860–7863 (2014).

6. Yang, J. *et al.* Two-Dimensional Hybrid Nanosheets of Tungsten Disulfide and Reduced Graphene Oxide as Catalysts for Enhanced Hydrogen Evolution. *Angew. Chem. Int. Ed.* **52,** 13751–13754 (2013).

7. Xu, Y., Wu, R., Zhang, J., Shi, Y. & Zhang, B. Anion-exchange synthesis of nanoporous FeP nanosheets as electrocatalysts for hydrogen evolution reaction. *Chem. Commun.* **49,** 6656–6658 (2013).

8. Long, X. *et al.* Metallic Iron–Nickel Sulfide Ultrathin Nanosheets As a Highly Active Electrocatalyst for Hydrogen Evolution Reaction in Acidic Media. *J. Am. Chem. Soc.* **137,** 11900–11903 (2015).

9. Kong, D., Wang, H., Lu, Z. & Cui, Y. CoSe2 Nanoparticles Grown on Carbon Fiber Paper: An Efficient and Stable Electrocatalyst for Hydrogen Evolution Reaction. *Journal of the American Chemical Society* **136,** 4897–4900 (2014).

10. Xu, Y.-F., Gao, M.-R., Zheng, Y.-R., Jiang, J. & Yu, S.-H. Nickel/Nickel(II) Oxide Nanoparticles Anchored onto Cobalt(IV) Diselenide Nanobelts for the Electrochemical Production of Hydrogen. *Angew. Chem. Int. Ed.* **52,** 8546–8550 (2013).

11. Popczun, E. J. *et al.* Nanostructured Nickel Phosphide as an Electrocatalyst for the Hydrogen Evolution Reaction. *Journal of the American Chemical Society* **135,** 9267–9270 (2013).

12. Siracusano, S. *et al.* An electrochemical study of a PEM stack for water electrolysis. *International Journal of Hydrogen Energy* **37,** 1939–1946 (2012).

13. Faber, M. S., Lukowski, M. A., Ding, Q., Kaiser, N. S. & Jin, S. Earth-Abundant Metal Pyrites (FeS2, CoS2, NiS2, and Their Alloys) for Highly Efficient Hydrogen Evolution and Polysulfide Reduction Electrocatalysis. *The Journal of Physical Chemistry C* **118,** 21347–21356 (2014).

14. Shalvoy, R. B. & Reucroft, P. J. Characterization of a sulfur- resistant methanation catalyst by XPS. *Journal of Vacuum Science & Technology* **16,** 567–569 (1979).

15. Binder, H. Die Anwendung der Röntgenphotoelektronenspektroskopie zur Klärung von Bindungsfragen in Eisen-Schwefelverbindungen. *Z. Naturforsch.* **28 b,** 255–262 (1973).

16. Biesinger, M. C. *et al.* Resolving surface chemical states in XPS analysis of first row transition metals, oxides and hydroxides: Cr, Mn, Fe, Co and Ni. *Applied Surface Science* **257,** 2717–2730 (2011).

17. Grosvenor, A. P., Biesinger, M. C., Smart, R. S. C. & McIntyre, N. S. New interpretations of XPS spectra of nickel metal and oxides. *Surface Science* **600,** 1771–1779 (2006).

18. Grosvenor, A. P., Kobe, B. A., Biesinger, M. C. & McIntyre, N. S. Investigation of multiplet splitting of Fe 2p XPS spectra and bonding in iron compounds. *Surface and Interface Analysis* **36,** 1564–1574 (2004).

19. Smart, R. S. C., Skinner, W. M. & Gerson, A. R. XPS of sulphide mineral surfaces: metal-deficient, polysulphides, defects and elemental sulphur. *Surface and Interface Analysis* **28,** 101–105 (1999).

20. Perdew, J. P., Burke, K. & Ernzerhof, M. Generalized Gradient Approximation Made Simple. *Phys. Rev. Lett.* **77,** 3865–3868 (1996).

21. Perdew, J. P., Burke, K. & Ernzerhof, M. Generalized Gradient Approximation Made Simple [Phys. Rev. Lett. 77, 3865 (1996)]. *Phys. Rev. Lett.* **78,** 1396–1396 (1997).

22. Adamo, C. & Barone, V. Toward reliable density functional methods without adjustable parameters: The PBE0 model. *The Journal of Chemical Physics* **110,** 6158–6170 (1999).

23. Schäfer, A., Horn, H. & Ahlrichs, R. Fully optimized contracted Gaussian basis sets for atoms Li to Kr. *The Journal of Chemical Physics* **97,** 2571–2577 (1992).

24. Neese, F. The ORCA program system. *WIREs Comput Mol Sci* **2,** 73–78 (2012).

25. TURBOMOLE V6.6 2014, a development of University of Karlsruhe and Forschungszentrum Karlsruhe GmbH, 1989-2007, TURBOMOLE GmbH, since 2007; available from http://www.turbomole.com.

26. Ahlrichs, R., Baer, M., Haeser, M., Horn, H. & Koelmel, C. Electronic structure calculations on workstation computers: The program system turbomole. *Chem. Phys. Lett.* **162,** 165 (1989).

27. Grimme, S., Antony, J., Ehrlich, S. & Krieg, H. A consistent and accurate ab initio parametrization of density functional dispersion correction (DFT-D) for the 94 elements H-Pu. *The Journal of Chemical Physics* **132,** 154104 (2010).

28. Eichkorn, K., Treutler, O., Oehm, H., Haeser, M. & Ahlrichs, R. Auxiliary basis sets to approximate Coulomb potentials. *Chem. Phys. Lett.* **240,** 283 (1995).

29. Eichkorn, K., Weigend, F., Treutler, O. & Ahlrichs, R. Auxiliary basis sets for main row atoms and transition metals and their use to approximate Coulomb potentials. *Theor. Chem. Acc.* **97,** 119 (1997).

30. Weigend, F. Accurate Coulomb-fitting basis sets for H to Rn. *Phys. Chem. Chem. Phys.* **8,** 1057 (2006).

31. Sierka, M., Hogekamp, A. & Ahlrichs, R. Fast evaluation of the Coulomb potential for electron densities using multipole accelerated resolution of identity approximation. *J. Chem. Phys.* **118,** 9136 (2003).
